# A conceptual framework for increasing clinical staff member involvement in general practice: a proposed strategy to improve the management of low back pain

**DOI:** 10.1186/s12875-019-0923-x

**Published:** 2019-02-21

**Authors:** Allan Riis, Emma L. Karran, Jonathan C. Hill, Martin B. Jensen, Janus L. Thomsen

**Affiliations:** 1 Center for General Practice at Aalborg University, Fyrkildevej 7, 9220 Aalborg, Denmark; 2 Sansom Institute for Health Research, University of South Australia, GPO Box 2471, Adelaide, South Australia 5001 Australia; 3 Arthritis Research UK Primary Care Centre, Research Institute for Primary Care & Health Sciences, Keele University, Staffordshire, ST5 5BG UK

**Keywords:** Implementation, Organizational change, General practice, Low back pain

## Abstract

**Background:**

Low back pain affects about 80% of all adults, many of whom consult general practice. Providing management can be challenging, in part due to the scarcity of effective treatment methods. There is broad consensus in international clinical practice guidelines to provide patients with information about the nature of their pain and recommend them to stay active despite discomfort. Delivering this information is time-demanding and challenged by the limited available resources in general practice in many countries. Furthermore, general practice settings are highly variable in size and in their composition of clinical staff members – which presents difficulties, but also opportunities for developing alternative approaches to clinical management. Expanding the patient consultation time by involving clinical staff members (aside from the general practitioner) has been found feasible for other conditions. We propose that this approach is applied for non-specific low back pain. Consequently, we suggest the involvement of clinical staff members as part of a new strategy for managing low back pain in general practice.

**Main text:**

Multifaceted implementation strategies have the potential to effectively enable change in the clinical management of patients with low back pain in general practice if they are based on theory and are tailored to stake holders. Inspired by the Medical Research Council’s guidance for complex interventions and the ChiPP (Change in professional performance) statement, we suggest applying the following two policy categories: organizational change (environmental/social planning) and service provision. This will involve attention to environmental restructuring, modelling, enabling, education, training, persuasion, and incentivising of general practices, with an over-arching strategy of involving clinical staff members in the management of low back pain.

**Conclusion:**

This is a pre-clinical proposal of a multifaceted strategy to support the delivery of evidence-based treatment for patients with low back pain in general practice. As an original idea, we suggest it would be feasible to involve clinical staff members in the delivery of information and advice to patients, whilst the general practitioner remains responsible for diagnostic decision-making.

## Background

The global burden of low back pain (LBP) is extensive; LBP has recently been identified to be the single leading cause of disability worldwide [1] and has an estimated lifetime prevalence of more than 80% [2]. The high prevalence of LBP leads to high healthcare utilization for this condition. In Denmark for example, it is the most frequent reason for patients consulting general practice [3].

In developed counties, the role of general practitioners (GPs) is typically to assess and triage patients presenting with LBP – aiming to identify and appropriately manage serious causes of LBP like fractures, cancer, infections, or inflammatory diseases such as axial spondylarthitis. If serious underlying disease is suspected, patients are likely to be referred for further investigations, specialist assessment and/or treatment [4]. In most cases however, the underlying causes of LBP remains unknown with only 1–5% of patients having serious underlying disease [4]. Consequently, it is usually most appropriate that patients with LBP are treated in primary care.

Primary care practice guidelines for the management of ‘non-specific’ LPB (i.e. LBP without any specific pathology identified) generally recommend a non-invasive and non-pharmacological approach. Practitioners are advised to (I) provide information about LBP and support of self-management strategies, (II) advice for patients to stay active, (III) consider other treatment approaches like exercise therapy or manual therapy, and (IV) avoid unnecessary referrals for further examinations, like scans and x-rays [4–8].

The average length of a GP consultation varies widely between countries: Germany (7.6 min); the United Kingdom (9.2 min); the Netherlands (10.2 min); and in Sweden it is estimated to exceed 20 min. However, a large proportion of the global population spend only a few minutes with their GP [9]. GPs have expressed frustration with the disparity between guideline-based recommendations for care, and the practicalities of implementing these recommendations in ‘real-world’ (time-constrained) situations [10]. In which, specialist services should be avoided in most cases and are difficult to access [10].

The GP may choose to refer the patient on to a physiotherapist or chiropractor for further information and self-management support. However, these supplementary treatment options frequently involve high individual patient costs or specific private employment contracts, and thus, are not suitable for all patients. Involvement of job centre or workplace personnel, referral to psychologists, or social councillors may also be worthwhile options to consider for some patients. Again, these options are only suitable for a limited number of patients. A newly published series in the Lancet highlights that the burden of low back pain is increasing and greater attention on this problem is needed [11]. In the series it is furthermore argued that the evidence points to the existence of promising solutions to positively improve LBP treatment through more focused strategies, e.g. by the redesign of clinical pathways [12].

**Fig. 1:**
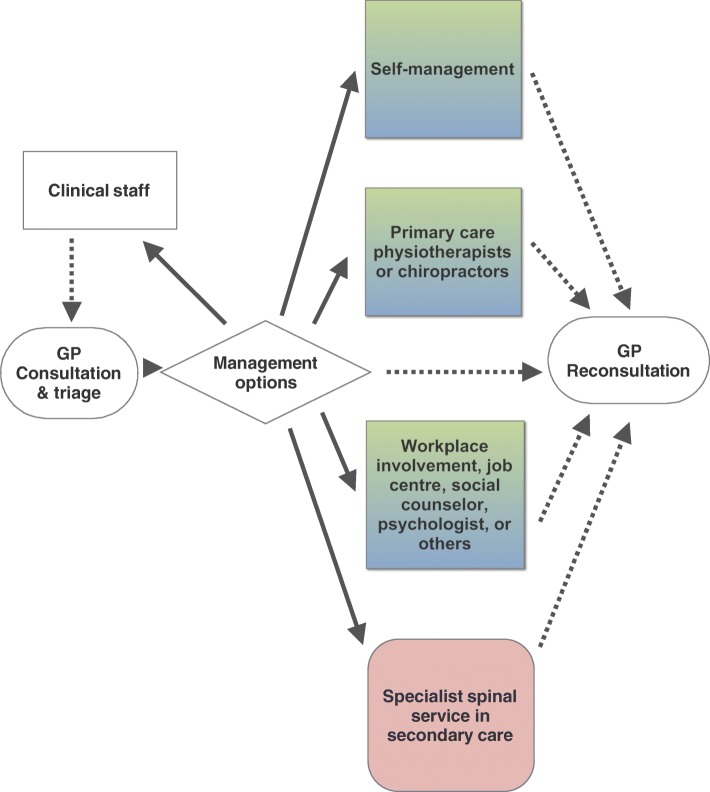
General practitioner’s management options for low back pain. *Legend:* General practitioners (GPs) management options for patients with low back pain. Involving clinical staff members is added to the existing treatment options in this model for general practice. In Denmark, GPs are gate keepers to other health care service in primary or secondary care (illustrated with solid lines). If the management option is not suitable or a change in symptoms is occurring other health care providers can refer back to the GP (illustrated with dotted lines)

A new pathway can include the involvement of clinical staff members, which may lead to extended total consultation time with patients. Furthermore, clinical staff members (e.g. practice nurses) are already experienced in motivating patients to increase their activity and integrate lifestyle changes to enhance self-management. Therefore, we believe that involving clinical staff members in the treatment of patients with LBP presents a new possibility for improving the clinical management of LBP patients who present to their general practitioner (Fig. 1). In this scenario – the patient undergoes an initial consultation with the GP who assesses the patient and (when indicated) confirms that there is a low likelihood of serious diseases as an underlying cause of the patient’s symptoms. Clinical staff members are then available to provide LBP-related information, support self-management, and even instruct patients in standard exercise or physical activity.

While this represents an ideal approach, it must be considered that some GP practices will be more suited to this type of organizational change than other GP practices. However, this reorganization is in line with current life-style interventions offered to patients with diabetes or heart diseases in general practice in many countries [13]. Nurses working on their own have demonstrated similar patient outcomes to those of doctors in the primary care management of some chronic diseases; delivering care that is acceptable, feasible, and sustainable [14, 15]. Furthermore, nurses working in general practice are able to reduce hospitalisation [13]. Practice nurses and other clinical staff members may therefore be considered adequate substitutes for GPs in treating some patients; and their expanded use in primary care clinics is likely to have cost-saving potential. Financial benefits will, however, depend on the differential salaries between doctors and the clinical staff members, and productivity considerations [15]. Moreover, patients express that their satisfaction with disease management is dependent on a collaborative relationship between GPs and nurses [14]. We, therefore, suggest a model in which GPs initially evaluate and diagnose the patient prior to engaging with the clinical staff who will subsequently manage the patients’ follow-up consultations. Formal delegation guidelines can describe and standardise these procedures to facilitate smooth process towards better primary care treatment of LBP and thereby reduce unnecessary referrals to specialist treatment. The aim of this paper is to propose a model for organizational change that involves clinical staff members in the routine management of patients with LBP in general practice.

### Main text

Previous studies have shown that interventions to support practitioners’ implementation of guideline-based management can change referral intentions [16], reduce referrals from general practice to secondary care, and reduce healthcare cost by £ − 93.20 per patient consulting general practice [17]. In the latter study from Denmark, the general practice stakeholders were involved in the development of the intervention components [18] and a stepwise method of model development was applied [19]. The intervention components were designed to address GPs capabilities, opportunities, and motivation to deliver evidence-based treatment of patients [20]. In this proposed model for organizational change, we suggest the application of a similar multifaceted theoretical approach to facilitate the integration of a complex intervention into general practice settings. In this proposal we have included elements, which are supported by the literature and we consider realistic to implement in general practice.

Multifaceted implementation strategies have been shown effective in improving care, but are not always more effective than simple strategies [21]. Promisingly, engaging stakeholder involvement in combination with applying multifaceted strategy has been found to be effective in improving health care [22] and is the strategy suggested in this proposal.

Our proposed ‘umbrella’ of implementation components require changes at the organizational level, GP level, clinical staff member level, and at the patient level. However, since the primary target behaviour is referral to secondary care by the GP and since the GP is the gatekeeper regarding this decision in many countries e.g. England and Denmark [23], we consider the GPs to be the focus of our model. We propose applying environmental (organizational) and service provision policies to facilitate this process [20]. These policies are important as they link directly with interventions that target the GPs capability, opportunity, and motivation for changing the management of patients with LBP with special attention to supporting GPs in decreasing referrals to specialist spinal service in secondary care. The intervention components included in this generalised model are considered to address the domains of guideline factors, individual health professional factors, professional interactions, patient factors, incentives and resources, and capacity for organisational change. These domains have been found important when improving healthcare practices [24]. General practices are, however, organised differently between and within countries. Consequently, it is important to acknowledge the need and importance of tailoring the model to different settings and/or specific patients [25]. It is not clear how best to tailor interventions [26]. However, when implementing this intervention in a specific setting, we suggest considerable effort is made to address local enablers and barriers for implementation (i.e. using the behaviour change wheel) [20]. Below, actions in the generalised model are grouped under the following subheadings; organisational change, general practice support and training, specific intervention components, and incentives (Table 1).

**Table 1:** Activities aimed at improving the treatment of patients with low back pain in general practice

Possible actions and elements for changing general practice behaviour	Capability (physical / psychological)	Opportunity (physical / social)	Motivation (reflective / automatic)
Organisational changes			
Knowledge of all components included in the umbrella of interventions	X		
Clinic staff delivery of standard information	X		
Clinical staff addressing psycho-social barriers for recovery	X		
Clinical staff instruction in standard exercise programmes	X		
Delegation guidelines	X		
General practice support and training			
Outreach visit with a reorganizational focus		X	X
Training sessions of clinical staff members delivered in the clinic		X	X
National annual clinical staff educational courses		X	X
Local GP Innovation Leaders		X	X
Cascade the proposed organizational changes in national journals and newsletters aimed at GPs and clinical staff members			X
Hotline service		X	
Audits by guideline facilitators		X	X
Specific intervention components			
Paper folders and online access to standard information		X	
Paper folders and online access to exercise programmes		X	
Cultural adaptation of folders and online material		X	
Access to screening tools		X	
Integration of intervention components within the GP medical record		X	
Intervention fidelity feedback to GPs		X	X
Incentives			
Greater clinical staff involvement			X
Extended consultation times			X
Greater equity in patient care		X	X
Addressing the frustration caused by missing medical treatment options			X

*Legend:* An umbrella of intervention components to address GPs capability, opportunity, and motivation to increase the uptake of low back pain guidelines in general practice and to decrease referral of patients with low back pain to specialist spinal service in secondary care

These components are included on the basis of the literature [24, 27]. A unique and novel aspect to our proposal is that for the first time these components have been combined into a useful primary care ‘tool box’ for the treatment of LBP. The components included in our implementation strategy are therefore multifaceted and described on a general level and we would highlight that some of the individual components may need to be adapted following an assessment of the specific national context and change opportunities [28].

### Identifying barriers and enablers to practice change

Since this is still at the study proposal stage, we have not yet studied and identified barriers and facilitators for each specific setting. To strengthen the development and implementation of the model, further investigation into stakeholders’ barriers and enablers to this change is needed. We therefore suggest integrating an iterative, qualitative approach that includes interviews, focus groups and semi-structures questionnaires to understand the perceptions of all key stakeholders and their acceptance of the proposed changes. Identified barriers and enablers then need to inform further intervention development. Important components to consider can be the availability of both paper and online versions of self-management information/material, delivery of outreach visits, the hotline service, and the application of online screening tools. All components will need to be tested separately, and then pilot tested combined in a multifaceted strategy in a clinical setting.

We acknowledge the challenges to widespread organizational change that are presented by varying (current) organizational structures, discrepant numbers of GPs and clinical staff members across clinics, and inconsistencies in available resources. Consequently, we suggest an umbrella of initiatives aimed at improving the clinical management of low back pain in a manner that can be tailored to individual settings. This will allow general practices to choose whether to fully engage with the model and include all proposed components or opt to integrate fewer components - with the potential to incorporate more components at a later stage. This flexibility, along with the development of positive and enduring partnerships with the health care providers is considered imperative to the successful implementation of sustainable practice change. Principally, intervention components must satisfy all key stakeholders, including the GPs, clinical staff, patients, and policy makers. In this initial proposal, we suggest seven types of intervention functions (Table 2).

**Table 2:** Applied intervention functions with examples of operationalization

Intervention function	Examples of operationalization
Environmental restructuring	Organizational change with clinical staff member involvement
Modelling	Cascading of the model through national journals and newsletters. Proposals for applied delegation guidelines
Education	National conferences. National annual clinical staff courses
Training	Outreach visits. Feedback (audits)
Enabling	Printed and online information material. Hotline service. Access to screening tools (for patients with pain > 2 weeks)
Persuasion	Local GP Innovation Leaders. Actively implementing this proposal as a method to manage the frustration with the limited existing treatment options
Incentivisation	Improving treatment through a long term investment for the GP practices

*Legend:* Suggested intervention functions to support a change in the management of patients with non-specific low back pain

In developing this model, we considered the MRC (A Framework for Developing and Evaluation of RCTs for Complex Interventions to Improve Health, 2000) framework together with the MRC (Developing and Evaluating Complex Interventions, 2008) update of the framework to provide overarching guidance for the development of evaluation of the intervention [29, 30]. The MRC framework is commonly used for developing complex interventions [31]. The framework recommends the use of iterative processes in the development of complex interventions, thereby allowing for loops of changes where information found in later phases can feed back to earlier phases. Meanwhile our organizational proposal is very much depending on GPs’ acceptance and willingness to engage in changing their management of patients with LBP. Therefore, we plan to incorporate a number of interventions directed at changing physician performance: the ChiPP (Change in professional performance) statement [19]. This statement provides a framework for developing, introducing, and reporting changes in professional behaviour in stages applied for traditional medical research [19]. When the model has reach an acceptable standard and feasibility of implementation has been determined, the multifaceted implementation strategy should be studied for effectiveness.

Neither the final content of the intervention (the umbrella of components) nor the detailed methodology for testing, efficacy studies, and effectiveness studies are provided in this proposal. The precise content and methodology will very much depend on further detailed protocol development as well as the input from stakeholders. The proposal in this paper outlines the content of our current, preclinical phase of model development. It also endeavours to address the challenges posed by developing a complex intervention, while at the same time striving to keep the story simple and to maintain transparency in the reporting of process. This is considered important to enable others to make use of future findings. We expect that some findings will be generalizable in nature and therefore able to inform healthcare delivery in other settings/countries, while other findings may be considered local adaptations without broader applicability. In Denmark, GPs are self-employed and work on contract for the public funder using a national agreement that details services and reimbursement based on a fee-for-service system [23]. In other countries, however, expenses for nurse salary might be a larger barrier for implementation. Furthermore, in other countries nurse practitioners virtually do not exist or have limited role in direct patient care.

### Possible effects of the proposed strategy

Previously implementation interventions outside of Denmark have shown modest effects and pointed to the need for increasing the duration of the interventions to obtain sustained effects [32, 33]. This proposed strategy, however, involves continuous education of clinical staff members and a sustained external support function, thereby optimising the potential for the effects of the intervention to be maintained. Through the promotion of more guideline concordant management, this intervention aims to reduce waste and unnecessary health service utilization and has the potential to result in improved patient outcomes. Since, training of new clinician groups does not require addressing unlearning of not guideline concordant procedures and the involvement of clinical staff members can expand the total clinician time with the patient. We believe this intervention can reduce healthcare related cost by up to 20% in countries with a primary care based system. This intervention is less relevant to countries in which patients can circumvent gate keeping by accessing specialist care directly.

## Conclusion

We propose a new strategy involving organizational change to support the implementation of current guidelines for treating LBP in general practice and to reduce referrals to specialist care service in secondary care.

## Abbreviations

GP: General practitioner
LBP: Low Back Pain
MRC: Medical Research Council
